# Clinical Management of Financial Toxicity–Identifying Opportunities through Experiential Insights of Cancer Survivors, Caregivers, and Social Workers

**DOI:** 10.3390/curroncol29100609

**Published:** 2022-10-14

**Authors:** Christopher J. Longo, Louisa G. Gordon, Rebecca L. Nund, Nicolas H. Hart, Laisa Teleni, Carla Thamm, Olivia Hollingdrake, Fiona Crawford-Williams, Bogda Koczwara, Tamara Ownsworth, Stephen Born, Sue Schoonbeek, Leanne Stone, Christie Barrett, Raymond J. Chan

**Affiliations:** 1 Health Policy and Management, DeGroote School of Business, McMaster University, Hamilton, ON L8S 4M1, Canada; 2 QIMR Berghofer Medical Research Institute, Brisbane, QLD 4006, Australia; 3 Cancer and Palliative Care Outcomes Centre, Queensland University of Technology, Brisbane, QLD 4059, Australia; 4 School of Nursing, Faculty of Health, Queensland University of Technology, Brisbane, QLD 4059, Australia; 5 School of Health and Rehabilitation Sciences, University of Queensland, Brisbane, QLD 4067, Australia; 6 Caring Futures Institute, College of Nursing and Health Sciences, Flinders University, Adelaide, SA 5001, Australia; 7 School of Medical and Health Sciences, Edith Cowan University, Perth, WA 6050, Australia; 8 Institute for Health Research, University of Notre Dame Australia, Perth, WA 6160, Australia; 9 Flinders Centre for Innovation in Cancer, Flinders Medical Centre, Flinders University, Adelaide, SA 5001, Australia; 10 School of Applied Psychology & the Hopkins Centre, Menzies Health Institute Queensland, Griffith University, Brisbane, QLD 8768, Australia; 11 Princess Alexandra Hospital, Metro South Health, Brisbane, QLD 4222, Australia

**Keywords:** cancer survivors, caregivers, financial burden, financial distress, financial toxicity

## Abstract

Perspectives of cancer survivors, caregivers, and social workers as key stakeholders on the clinical management of financial toxicity (FT) are critical to identify opportunities for better FT management. Semi-structured interviews (cancer survivors, caregivers) and a focus group (social workers) were undertaken using purposive sampling at a quaternary public hospital in Australia. People with any cancer diagnosis attending the hospital were eligible. Data were analysed using inductive-deductive content analysis techniques. Twenty-two stakeholders (*n* = 10 cancer survivors of mixed-cancer types, *n* = 5 caregivers, and *n* = 7 social workers) participated. Key findings included: (i) genuine concern for FT of cancer survivors and caregivers shown through practical support by health care and social workers; (ii) need for clarity of role and services; (iii) importance of timely information flow; and (iv) proactive navigation as a priority. While cancer survivors and caregivers received financial assistance and support from the hospital, the lack of synchronised, shared understanding of roles and services in relation to finance between cancer survivors, caregivers, and health professionals undermined the effectiveness and consistency of these services. A proactive approach to anticipate cancer survivors’ and caregivers’ needs is recommended. Future research may develop and evaluate initiatives to manage cancer survivors and families FT experiences and outcomes.

## 1. Introduction

Financial toxicity (FT) describes the objective financial burden and subjective financial distress from cancer and its treatment [1,2,3]. A systematic review of 25 studies involving 271,732 cancer survivors [3] reported one in three cancer survivors experiencing significant FT from direct costs (e.g., out-of-pocket expenses) and two in three from indirect costs (e.g., loss of income), applicable to user-pay and universal healthcare systems [3,4]. In Australia, healthcare is provided in both the public and private settings. While patients receiving private care pay higher out-of-pocket costs for treatment-related expenses, direct costs of cancer and its treatment can apply to patients receiving private (e.g., care and treatment, imaging, tests) and public care (e.g., accommodation, parking, supportive care). Depending on a cancer survivor’s (and family’s) circumstance, the response to FT may include the use of savings, sale of assets, borrowing money, or reduced adherence to treatment affecting cancer survivors’ health-related quality of life [5,6], and increasing their psychological and physical symptom burden [7,8,9,10].

Minimising the full impact of FT requires responses at an individual, interpersonal, and health system level, though early assessment and intervention is necessary to identify and alleviate the impact of FT on cancer survivors prior to commencement, during, and after treatment [11,12]. These interventions may include informing cancer survivors of financial implications of treatment options in a timely manner [11,13]; avoiding low-value treatment or care [13]; advocating for cancer survivors in employment or accessing financial aid [13]; empowering cancer survivors through a return-to-work plan [14]; and managing distress associated with financial hardship [15].

Many qualitative studies have examined the experiences of cancer survivors’ FT [16], however, these studies focus on the nature of impact and experiences of FT itself, and not the clinical interventions offered by their health care professionals (HCPs) for managing or alleviating FT. Understanding FT management experiences and expectations would be helpful to inform: (1) immediate service improvement in the clinical setting [17,18]; and (2) development of a cancer survivor-reported experience measure, specifically in relation to FT management.

Perspectives of cancer social workers (SWs) on FT management also provide important insights due to their day-to-day role supporting cancer survivors in relation to their financial and social wellbeing [19,20]. Our study is not an evaluation of the service provided by any single discipline, but rather an exploration of the viewpoints of cancer survivors, caregivers and SWs on FT management at the clinical level. In this study, we adopt the most widely used definition of cancer survivors, as any individuals with cancer from the time of diagnosis until the end of life [21]. Our aims were to identify the perspectives of cancer survivors, caregivers and SWs on the interactions related to managing FT and to identify opportunities for better management of FT.

## 2. Materials and Methods

A qualitative descriptive methodology was used to describe key stakeholders perspectives of the clinical management of FT. Qualitative descriptive studies draw from naturalistic inquiry and involve the study of real-world situations as they occur naturally without any pre-determined theories or frameworks [22,23]. Consolidated criteria for reporting qualitative data research (COREQ) guidelines were adopted [24].

### 2.1. Participants

Individuals with any cancer diagnosis at any point across the cancer trajectory (i.e., diagnosis, during treatment (including palliative intent), or post-treatment) attending the participating cancer centre, who expressed any financial difficulties or concerns, were eligible. Participants were referred by health professionals (medical, nursing and allied health) at the cancer centre. Survivors who were too unwell to participate or in the last weeks or days of life were excluded. Survivors who did not speak English were included where an interpreter could be arranged, however, this was not required. All social workers who worked with people with cancer were invited by the researchers via email. Those who were employed at the hospital and cared for cancer survivors for at-least 12 months were eligible. Sample size was determined pragmatically as all potential participants who expressed interest in the study over a three-month period were included. The purposive approach to sampling ensured a diversity within the sample across cancer types, public or private health insurance and experiences. A total of twenty-two participants completed the study (*n* = 10 cancer survivors of mixed-cancer types, *n* = 5 caregivers, and *n* = 7 social workers). It was expected that this selective and well-informed sample would provide rich insights [25], and could achieve thematic saturation based on our past experiences [26].

### 2.2. Setting

The study was conducted at the Princess Alexandra Hospital within Metro South Hospital and Health Services (Brisbane, QLD, Australia). Cancer Services at the participating centre provide medical oncology, radiation oncology, and hematology inpatient and ambulatory care, which is mainly publicly funded, thus a minority receiving private care. Some cancer survivors received private care in other health services prior to receiving state-funded care at this hospital. These experiences are also captured and within scope in exploring the research questions. Ethical approval was granted by the Metro South Hospital and Health Services Human Research Ethics Committee (MSHHS HREC/2018/QPA/249).

### 2.3. Procedure

*Cancer survivors and caregivers*—Semi-structured interviews were conducted between July–November 2018 with cancer survivors and their caregivers to explore their views concerning FT management in their care experience. Written informed consent was obtained prior to data collection. Interview guides for survivors and caregivers were designed by the research team with consumer input (see Appendix A and Appendix B). Interviews were conducted face-to-face or via the telephone by a nurse investigator (SB/RJC) with experience and training in qualitative interviewing. Interviews ranged from 20 to 60 min and were audio-recorded, transcribed verbatim and checked for accuracy. Transcripts were returned to participants to for confirmation or correction.

### 2.4. Procedure

*Social workers*—A focus group was conducted with SWs on 22nd January 2019. Video-recorded, face-to-face, semi-structured focus groups were conducted by a nurse investigator (RJC), which ran for approximately 90 min. Informed consent was obtained prior to the focus group where SWs were informed that this study was not an evaluation of the SW service. Rather, it aimed to explore their perceptions of FT management internal or external to the local cancer service, including all disciplines involved in previous and current care. SWs were asked for their insights via two guiding questions: (1) what positive and negative experiences of cancer survivors and caregivers have you observed in relation to clinical management of FT? and (2) what are the opportunities for improvement in terms of clinical management of FT for cancer survivors and their caregivers?

### 2.5. Data Analysis

Two researchers (CJL and RJC) engaged with and analysed the data using inductive-deductive content analysis techniques [27] to identify the perspectives of cancer survivors, caregivers, and SWs on the management of FT and suggestions for service improvement [17,18]. These techniques encourage analysis to be iterative and ongoing, and commence from the beginning of data collection [28]. During data collection, interview summaries and notes were made following each interview that allowed exploration of important ideas that arose in preceding interviews and facilitated early and ongoing interpretations of the data. A process of in-depth reading and re-reading of the data, with notes and identifiers written throughout the transcripts was used to determine themes. Coding was also undertaken by two authors (CJL and RJC) following conventional methods [27]. Coding units were developed into a master list and amalgamated into overall themes with additional themes added to the list as needed. Two authors (CJL and RJC) met to consolidate the perspectives, reach agreement on divergent cases, and revise the themes to ensure the full range of feedback for all stakeholders was included in the final analysis.

## 3. Results

**Figure 1:**
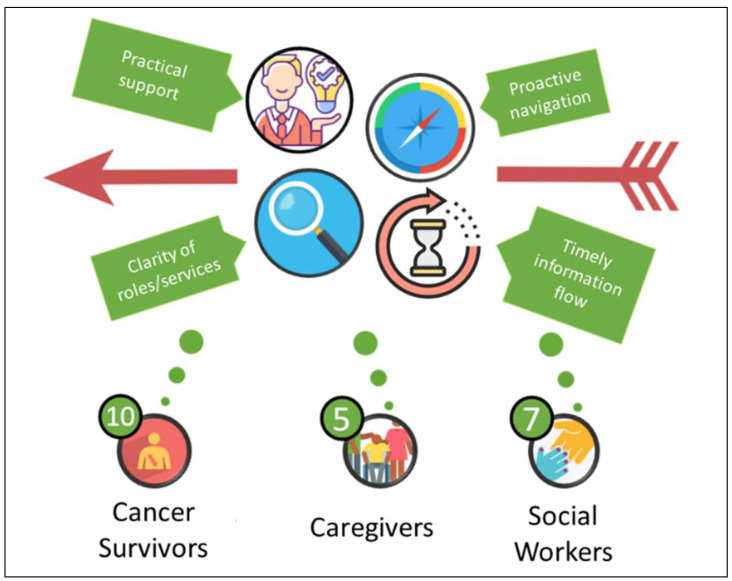
According to cancer survivors, caregivers, and social workers, quality financial toxicity management should include: (i) genuine concern for financial toxicity of cancer survivors and caregivers through practical support by health care providers; (ii) clarity of role and services; (iii) timely information flow; and (iv) proactive financial navigation.

Ten cancer survivors, five caregivers, and seven SWs participated. All participants were <80 years of age, with higher female representation, full representation for education and income, and a variety of tumor types (Table 1 and Table 2). Most survivors (8/10) did not have private health insurance. One had a private insurance plan, and one was not an Australian citizen and did not have universal health coverage. SW participants were 26–65 years of age, all female, and represented early to seasoned experience in social work and cancer care settings (Table 2). Findings were categorised into four key themes (Figure 1): (i) genuine concern through practical support (ii) need for clarity of roles and services; (iii) importance of timely information flow; and (iv) proactive navigation as a priority.

### 3.1. Genuine Concern through Practical Support

Many participants felt that some HCPs, particularly SWs, showed a genuine concern for the financial well-being of cancer survivors and were very supportive around financial issues.

“I think there’s a lot of help from doctors, from nurses and from SWs… I think they tried their best to provide that help, not only financially but emotionally and in many other aspects”. (Survivor #6).

Specifically, cancer survivors and caregivers perceived that SWs often made concerted efforts to ensure practical access to relevant resources and appropriate information.

“I expressed my concerns in terms of my finances. So, she of course offered guidance in that sense like, look, ‘if you need any documents for Centrelink to speed up the process, …we can find financial help, we can get a voucher for you at some point if you struggle too much for food’, … so she started talking about those options in that moment”. (Survivor #6).

“She (SW) was really helpful and … she got everything happening to get us the finances by the time I came and checked in for my operation”. (Survivor #5).

SWs provided practical, on-the-spot support to help survivors and their caregivers plan and manage financial issues as they emerged.

“I used my car. One thing (SW) was helping us with—the voucher for the petrol. It was $200 and it was quite a bit of help”. [Survivor #4].

“(SW)’s been really awesome, … if we needed anything financially, … there were times when she gave us vouchers for food and petrol, and they helped so many times. Like last Christmas we got some food vouchers and it meant that we could have Christmas lunch”. (Caregiver #4).

### 3.2. Need for Clarity of Roles and Services

Participants reported a lack of synchronised, shared understanding of roles in relation to finance between cancer survivors, caregivers, and HCPs. Although participants identified that HCPs showed concern and gave practical support, they suggested that at times, identifying and connecting with a HCP who could assist with financial matters proved challenging in a busy hospital environment. Even if patients and caregivers were given the correct person to talk to, one participant recognised this was not always prioritised.

“…we did try to contact the SW when I first met her here. But for whatever reason … my mum tried ringing because my mum came up from Victoria. But for whatever reason, she wasn’t getting the messages or whatever. “(Survivor #2).

SWs also suggested that their role in financial support was often not clearly understood by cancer survivors and caregivers.

“Sometimes I don’t think (patients) know that things may escalate along the pathway. Sometimes I think it’s even just an education issue around what the role could be for a SW to provide them information or where to start really”. (SW Focus Group).

This role confusion extended to front-line HCPs who were not always aware of the SW role and may not have the resources and time to identify or address the cancer survivors’ and caregivers’ financial burden themselves.

“Unless it’s overtly obvious that they’re [cancer survivors receiving private care] needing social work in that clinic, i.e., they’re really distressed, teary, family distressed or whatever, I get asked to come in but otherwise I am just not informed, I don’t see them, I see the public patients”. (SW Focus Group).

### 3.3. Importance of Timely Information Flow

Participants identified that the timeliness of financial support interventions is critical as delayed identification of financial issues may result in lost income or financial crisis, and subsequent distress. A SW participant articulated:

“…by the time they are referred, which can be 6, 8, 12 months down the track, they’re in a crisis situation financially... Alternatives that we could have explored and wouldn’t have potentially got in as much to crisis point”. (SW Focus Group).

Absence or delayed discussions about expected and unexpected costs especially from previous private care were highlighted by participants as a cause of distress.

“… it was a bit of a shock and a bit of an insult. I hadn’t really prepared for those sorts of costs…. when I went to see the medical oncologist initially, …, he charged $490. I did not see that coming”. (Survivor #1).

Earlier discussions with cancer survivors and their caregivers about upcoming costs would allow them to plan. Many mentioned the need for support in their applications for accessing Centrelink payment schemes.

“I’d just say if people could… be a bit more upfront about some of the costs for - it’s just part of the package. It’s part of the deal. Yes, you’ll debulk my tumor in my head and this is how much it’s going to cost, and these are all the other costs. It’d be nice to have some extra assistance with things” (Caregiver #1).

Often the onus was placed on survivors and caregivers to seek out information about services costs.

“He (cancer survivor) will ask them about the financial side of it… That should be quite plain and should be given to you… He’s always been doing that as well, but he wasn’t given that ability with his private medical oncologist and that experience really upset him and it really upset me”. (Caregiver #1).

“It would have been good if we’d been told about the carer’s payment a little bit earlier” (Caregiver #3).

### 3.4. Proactive Navigation as a Priority

Participants identified that it would be helpful for HCPs to anticipate cancer survivor and their caregiver needs through a proactive approach to financial navigation.

“Some people… might not need financial help. But those that do, someone that knows what’s available and also make the process a little bit easier, because we’ve got so much going on in our head with our treatment, our-trying to get back on our feet”. (Survivor #2).

The SW participants identified the importance of clarity around treatment pathways for patients and caregivers to help them plan and navigate their cancer journey and the financial implications of this.

“I think sometimes a clear treatment plan like because sometimes when they’re talking about treatment, they will talk about the initial treatment not the entire… But if you had known the whole time, back then, what your treatment plan was going to be like you’d probably plan it different financially”. (SW Focus Group).

Regular evaluations of patient’s finances along the treatment journey were also seen as an important intervention to manage financial burdens early and avoid crisis.

“The main thing would be, does the person…have access to the essential services, telephone, electricity, all the things that they need when they are at home? Because if they’re falling behind with those bills, obviously no telephone you get in strife, you can’t ring for an ambulance or the doctor to make an appointment”. (Survivor #2).

Furthermore, supporting the management of complex forms and accessing services early and at regular intervals was identified as important for managing FT.

“It would have been nicer if somebody had come to me and said right, these are the forms that you need … I’ve already had the doctor fill out the forms for you, so he’s filled out your part. There’s your part, your forms, you fill out and put in when you’re ready”. (Caregiver #4).

Providing a list of question-prompts for cancer survivors and caregivers to ask relevant HCPs at each decision point was posited as way to facilitate early information provision and to empower patients to navigate the system to meet their financial needs.

“I think probably a set of questions that you need to be asking at each decision point in the process with treating clinicians, doctors or whatever as far as financial services and things like that”. (Survivor #1).

Participants identified other opportunities to improve support for financial navigation in the acute care environment. Firstly, HCPs can act as advocate on behalf of cancer survivors in dealings with employers. “Maybe there needs to be some kind of working together with an employer to do a return-to-work plan”. (Caregiver #3). Secondly, HCPs can provide information concerning home support or care that falls outside of the hospital.

“… you need to have skilled staff, whoever that is, to be able to do the report medically or for income protection or whatever they’re needing to lodge, if they know that’s the case at the point that they’re told that, you’ll need six weeks off or six months off”. (SW Focus Group).

Thirdly, HCPs could work to reduce unnecessary face-to-face clinic visits, in turn reducing travel costs and missed workdays.

“If somebody gave you a phone call… it would save me coming in, which would save a problem for parking, because I wouldn’t be here”. (Caregiver #2).

## 4. Discussion

Although the nature and experiences of FT have been previously examined [4,10,16], our paper uniquely and explicitly focused on FT management at the clinical level. Participants were recruited in a public hospital; and some participants offered insights about care offered by public and external private settings. It is important to acknowledge that there is a group of cancer survivors who may switch between private and public systems across their cancer journey; and that FT occurs in a variety of health systems and settings [3,4]. Our findings suggested four areas for further attention in FT management: (i) genuine concern through practical support (ii) need for clarity of roles and services; (iii) importance of timely information flow; and (iv) proactive navigation as a priority.

A positive experience identified by cancer survivors was the presence of ‘genuine concern’ that most physicians, nurses, and SWs showed for their financial wellbeing, and the practical support offered by SWs. While cancer survivors and caregivers received financial assistance and support, the lack of clarity of roles and services shared between survivors, caregivers, and HCPs undermines the effectiveness and consistency of these services. Such findings indicate the need to develop consensus on roles and responsibilities in the clinical management of FT. In alignment with previous reports [11,29], some cancer survivors and caregivers perceived they were not always well-informed of the financial implications of their care. As such, many cancer survivors and caregivers experienced distress as they encountered unexpected costs from health services. To address this, Cancer Council Australia recently developed a standard for informed financial consent, outlining clear responsibilities of HCPs and the underpinning principles to ensure clear communication between HCPs and cancer survivors, transparency of treatment benefits and transparency of fees and costs associated with treatments [30]. Further work is required to encourage uptake of such standards and evaluate changes in behaviours and outcomes.

One barrier to financial care management identified by all participant groups was the lack of systematic, timely, and comprehensive financial information provision. Although financial and income support did occur for some, the support often came too late when financial issues had already arisen. Financial problems could escalate quickly and become dire if not managed early. Advanced notice of upcoming expenses would give cancer survivors and caregivers the opportunity to plan and possibly avoid or minimise some of the impacts experienced. Zhu and colleagues [16] highlighted that a lack of financial discussions often led to unpreparedness and distress. Consistent with their work [16], cancer survivors and caregivers in our study agreed a question prompt list at key decision points would aid a timely discussion about financial issues, allowing HCPs to identify those at risk of FT and facilitate earlier deployment of navigation support or assistance with income replacement forms.

While early discussion and comprehensive information provision are desirable, resources are finite in a stringent environment. Accordingly, it would be helpful for the multidisciplinary cancer team to reach consensus to inform the minimal financial care standards for all cancer survivors, with allocation of resources to those who are at higher risk of developing FT. Our recent longitudinal study of 391 breast cancer survivors demonstrated that certain clinical and demographic characteristics can predict survivors’ FT risk profile [7]. Such risk profiles are helpful to inform resource allocation. In addition, given that routinely collected survivor-reported outcome measures in the wider cancer care setting have clinical benefits across a number of cancer cohorts [31], it would be prudent to consider how such successes can be leveraged to enable appropriate FT screening and subsequent clinical actions [7]. Another approach to identify people requiring additional support may be the use of routine finance-related reported outcomes (FROMs) [32].

Participants provided two suggestions for further exploration. Firstly, financial distress may be lessened if stronger advocacy from HCPs and support workers is provided, especially for income replacement, government support, and social welfare (i.e., Centrelink) and negotiation with their employers. The role of financial navigators is not a new concept and has been implemented in the United States [33,34,35]. Such roles have a focus of easing financial hardship through reducing potential out-of-pocket costs and managing other financial needs including employment, food and housing [33,34,35]. Monak and colleagues [34] highlighted the importance of a strong, collaborative relationship between these financial navigators, SWs, care coordinators, and community services. Secondly, reducing unnecessary clinic visits may be a potential strategy to reduce costs associated with travelling or missed workdays. Such a strategy has been recognized as a driver for appropriate use of telehealth appointments as well as shared-care models involving cancer survivors’ general practitioners, which is increasingly important particular in the post-COVID era [36,37].

As this work was undertaken at a single Australian cancer centre, it is difficult to ascertain whether the themes would be consistent with other jurisdictions within Australia. Despite this limitation, little research has been conducted to specifically explore the perspectives of cancer survivors, caregivers, and SWs on the clinical FT management in Australia, providing direction to guide future research and improvement efforts. Second, this study was conducted prior to the COVID-19 pandemic, and therefore does not provide insights into specific care concerns that were heightened during the pandemic. Nevertheless, the emerged themes are generic in nature and should be applicable regardless of the type of financial concerns pre-, during and post-pandemic. Further research should explore FT-related care and management expectations in the post-COVID-19 era.

## 5. Conclusions

A consensus approach to provide clarity on roles and services in relation to FT management available in cancer centres is required. Earlier and more comprehensive information related to financial expenditures and income replacement is needed. Other opportunities may include timely financial discussion, systematic assessment of people at higher risk of developing FT, use of question-prompt list, potential role of financial navigators, and the potential of enhancing HCPs’ support roles including acting as advocates, and reduction of unnecessary appointments.

## Figures and Tables

**Table 1:** Characteristics of the cancer survivor and caregiver participants.

	Cancer Survivor (*n* = 10)	Caregiver (*n* = 5)
	*N* (%)	*N* (%)
Age 40–49 50–59 60–69 70–79	3 (30) 4 (40) 1 (10) 2 (20)	3 (60) 1 (20) 0 1 (20)
Sex Female Male	6 (60) 4 (40)	4 (80) 1 (20)
Diagnosis Central Nervous System Breast Head and Neck Genitourinary Leukaemia	1 (10) 2 (20) 2 (20) 2 (20) 3 (30)	N/A
Partnered versus non-partnered Partnered Non-Partnered	4 (40) 6 (50)	4 (80) 1 (20)
Highest qualification Lower than high school Completed high school Completed vocational training Completed university degree and above	3 (30) 2 (20) 4 (40) 1 (10)	1 (20) 2 (40) 0 2 (40)
Currently working for pay Yes No	4 (33) 8 (66)	3 (60) 2 (40)
Household Income (in AUD) ≤29,999 30,000–49,999 50,000–69,999 70,000–89,999 ≥90,000 Prefer not to say	1 (10) 2 (20) 2 (20) 2 (20) 2 (20) 1 (10)	N/A

Abbreviations: AUD, Australian Dollars; N/A, not applicable.

**Table 2:** Characteristics of the Social Workers.

	Social Worker (*n* = 7)
	*N* (%)
Age 26–35 36–45 46–55 56–65	2 (29) 2 (29) 2 (29) 1 (14)
Sex Female	7 (100)
Years of Experience in Social Work <5 years 5–10 years 11–20 years	2 (29) 0 5 (71)
Years of Experience in Cancer Care <5 years 5–10 years 11–20 years	3 (43) 3 (43) 1 (14)

## Data Availability

No data will be shared publicly as it may inadvertently reveal participants’ identity. However, datasets generated or analysed for this study are available from the corresponding author upon reasonable request.

## Appendix A. Interview Guide (Patient Version)

1. Tell me about any financial issues you have experienced since the diagnosis of your cancer/since your cancer have come back/since the diagnosis of your most recent cancer?

Potential subsequent prompts (if applicable):
  - Describe your experience and your feelings associated with these issues/events
  - How did this affect you, your partner, carer or your family?
  - Did you have any finance-related concerns with regards to your cancer treatment? If so, what were they?
  - Could you comment on your access to insurance and financial services (i.e., dealings with insurance and insurers, access to financial services, financial advice, loans etc.)
  - What guidance or assistance did you receive (and from who)? Were they useful? If not, why not?
  - What assistance, care or guidance would have been useful for the circumstance(s) you described above?
  - At what point of time would be useful for you/your carers to receive them?
  - Do you have any preference with regards to who you would like to receive the assistance or guidance from?

2. Have you had any employment issues/loss of income since the diagnosis of your cancer/since your cancer have come back/since the most recent diagnosis of your recent cancer?

Potential subsequent prompts (if applicable):
  - How have these issues affected you, your partner, carer or your family/loved ones, your employers and your colleagues? In what way?
  - Did you have a return-to-work plan? Tell me more about the plan.
  - Have you experienced any challenges in returning to work? What were these challenges? And how have you gone about to overcome these challenges?
  - Were there any symptoms or health concerns that restrict your ability to perform your duties at work? If so, what were they?
  - What assistance, care or guidance would have been useful for the circumstance(s) you described above?
  - At what point of time would be useful for you/your carers to receive them?
  - Do you have any preference with regards to timing and who you would like to receive the assistance or guidance from?
  - With all the issues/costs discussed above, were there any issues that are not specific to your cancer diagnosis or treatment? (i.e., you believe you would be experiencing that anyway with or without your cancer diagnosis)

3. Have you had any out-of-pocket costs for your cancer diagnosis and treatment?

Potential subsequent prompts:
  - Throughout the course of your illness, what were the expected and unexpected costs related to your cancer and cancer treatment?
  - Consider medications, GP, hospitalisations, medical tests, parking, transport, accommodation, home/selfcare assistance, medical equipment (name specific depending on care type) and supplies, home modification, health foods, complementary or alternative medicine, childcare
  - Did you require any travel and/or accommodation arrangements? How did you navigate through that process? Was any assistance provided to you? Was it useful? If not, why not?
  - With all the issues/costs discussed above, were there any issues that are not specific to your cancer diagnosis or treatment? (i.e., you believe you would be experiencing that anyway with or without your cancer diagnosis)

4. Is there anything you would like to add to what we have discussed so far.

## Appendix B. Interview Guide (Carer Version)

* Insert the patient’s name where applicable.

1. Tell me about any financial issues you have experienced since the patient’s diagnosis of your cancer/since the patient’s cancer have come back/since the diagnosis of the patient’s most recent cancer?

Potential subsequent prompts (if applicable):
  - How did this affect the patient, yourself, or your family?
  - Did you have any finance-related concerns with regards to the patient’s cancer treatment? If so, what were they?
  - Could you comment on your experience with regards to accessing insurance and financial services for the patient or your family? (i.e., dealings with insurance and insurers, access to financial services, financial advice, loans etc.)
  - Have you/did you receive any guidance or assistance did you or the patient receive (and from who)? Were they useful? If not, why not?
  - What assistance, care or guidance would have been useful for the circumstance(s) you described above?
  - At what point of time would be useful for the patient or you to receive them?
  - Do you have any preference with regards to who you would like to receive the assistance or guidance from?

2. Have you had any employment issues/loss of income since the patient’s diagnosis of your cancer/since the patient’s cancer have come back/since the diagnosis of the patient’s most recent cancer?

Potential subsequent prompts (if applicable):

- How have these issues affected the patient, or your family/loved ones, your employers and your colleagues? In what way?
- Have you experienced any challenges in your own work? What were these challenges? And how have you gone about to overcome these challenges?
- How did caring for the patient limit your ability work? Tell me more about that.
- What assistance, care or guidance would have been useful for the circumstance(s) you described above?
  At what point of time would be useful for you/your carers to receive them?
- Do you have any preference with regards to timing and who you would like to receive the assistance or guidance from?
- With all the issues/costs discussed above, were there any issues that are not specific to the patient’s cancer diagnosis or treatment? (i.e., you believe you would be experiencing that anyway with or without the patient’s cancer diagnosis)

3. Have you had any out-of-pocket costs for the patient’s cancer diagnosis and treatment?

Potential subsequent prompts:
  - Throughout the course of the patient’s illness, what were the expected and unexpected costs related to the patient’s cancer and cancer treatment?
    ◦ Consider medications, GP, hospitalisations, medical tests, parking, transport, accommodation, home/selfcare assistance, medical equipment (name specific depending on care type) and supplies, home modification, health foods, complementary or alternative medicine, childcare
  - What were the travel and/or accommodation arrangements? How did you navigate through that process? Was any assistance provided to the patient or yourself? Was it useful? If not, why not?
  - With all the issues/costs discussed above, were there any issues that are not specific to the patient’s cancer diagnosis or treatment? (i.e., you believe you would be experiencing that anyway with or without the patient’s cancer diagnosis)

4. In light of what have been discussed, is there any care you believe would very helpful for carers? What are they?
5. Is there anything you would like to add to what we have discussed so far. What are they?
